# Management of sulfonylurea‐treated monogenic diabetes in pregnancy: implications of placental glibenclamide transfer

**DOI:** 10.1111/dme.13388

**Published:** 2017-06-13

**Authors:** M. Shepherd, A. J. Brook, A. J. Chakera, A. T. Hattersley

**Affiliations:** ^1^ Institute of Biomedical and Clinical Science University of Exeter Medical School Exeter UK; ^2^ Exeter NIHR Clinical Research Facility Royal Devon and Exeter NHS Foundation Trust Exeter UK; ^3^ Lancashire Women and Newborn Centre Burnley General Hospital, East Lancashire NHS Hospitals Trust Burnley UK; ^4^ University of Manchester Manchester UK; ^5^ Royal Sussex County Hospital, Brighton and Sussex University Hospitals Brighton UK

## Abstract

The optimum treatment for HNF1A/HNF4A maturity‐onset diabetes of the young and ATP‐sensitive potassium (K_ATP_) channel neonatal diabetes, outside pregnancy, is sulfonylureas, but there is little evidence regarding the most appropriate treatment during pregnancy. Glibenclamide has been widely used in the treatment of gestational diabetes, but recent data have established that glibenclamide crosses the placenta and increases risk of macrosomia and neonatal hypoglycaemia. This raises questions about its use in pregnancy. We review the available evidence and make recommendations for the management of monogenic diabetes in pregnancy. Due to the risk of stimulating increased insulin secretion *in utero*, we recommend that in women with *HNF1A/ HNF4A* maturity‐onset diabetes of the young, those with good glycaemic control who are on a sulfonylurea per conception either transfer to insulin before conception (at the risk of a short‐term deterioration of glycaemic control) or continue with sulfonylurea (glibenclamide) treatment in the first trimester and transfer to insulin in the second trimester. Early delivery is needed if the fetus inherits an *HNF4A* mutation from either parent because increased insulin secretion results in ~800‐g weight gain *in utero*, and prolonged severe neonatal hypoglycaemia can occur post‐delivery. If the fetus inherits a K_ATP_ neonatal diabetes mutation from their mother they have greatly reduced insulin secretion *in utero* that reduces fetal growth by ~900 g. Treating the mother with glibenclamide in the third trimester treats the affected fetus *in utero*, normalising fetal growth, but is not desirable, especially in the high doses used in this condition, if the fetus is unaffected. Prospective studies of pregnancy in monogenic diabetes are needed.

## What's new?

- Recent data show that glibenclamide crosses the placenta and its use in pregnancy is associated with increased birth weight and neonatal hypoglycaemia. This has implications for the treatment of pregnant women with monogenic diabetes whose diabetes is usually well controlled with sulfonylureas.
- Optimum management of HNF1A/HNF4A maturity‐onset diabetes of the young (MODY) in pregnancy requires excellent glycaemic control in the first trimester to minimize the risk of fetal malformations, whilst avoiding the negative impact of glibenclamide on fetal weight gain in the third trimester.
- In mothers with ATP‐sensitive potassium channel (K_ATP_) neonatal diabetes, glibenclamide treatment in pregnancy can be beneficial if the fetus is affected because restoration of fetal K_ATP_ function will result in improved fetal growth.
- If the genotype of the fetus is unknown, when a parent has K_ATP_ neonatal diabetes or *HNF4A* MODY, serial antenatal ultrasound assessment of fetal growth may be used as a proxy to aid management decisions.
- Management of monogenic diabetes during pregnancy could be revolutionized in future by testing cell‐free fetal DNA in the mother.

**What's new?**

- Recent data show that glibenclamide crosses the placenta and its use in pregnancy is associated with increased birth weight and neonatal hypoglycaemia. This has implications for the treatment of pregnant women with monogenic diabetes whose diabetes is usually well controlled with sulfonylureas.
- Optimum management of HNF1A/HNF4A maturity‐onset diabetes of the young (MODY) in pregnancy requires excellent glycaemic control in the first trimester to minimize the risk of fetal malformations, whilst avoiding the negative impact of glibenclamide on fetal weight gain in the third trimester.
- In mothers with ATP‐sensitive potassium channel (K_ATP_) neonatal diabetes, glibenclamide treatment in pregnancy can be beneficial if the fetus is affected because restoration of fetal K_ATP_ function will result in improved fetal growth.
- If the genotype of the fetus is unknown, when a parent has K_ATP_ neonatal diabetes or *HNF4A* MODY, serial antenatal ultrasound assessment of fetal growth may be used as a proxy to aid management decisions.
- Management of monogenic diabetes during pregnancy could be revolutionized in future by testing cell‐free fetal DNA in the mother.

## Introduction

The recognition of monogenic diabetes is important as therapy can be improved accordingly. For several subtypes of monogenic diabetes, insulin treatment or other glucose‐lowering medication can be replaced by sulfonylureas. The common subtypes of maturity‐onset diabetes of the young (MODY) resulting from mutations in *HNF1A* and *HNF4A* are optimally treated with low‐dose sulfonylureas 1, 2 and, in the most common subtypes of permanent neonatal diabetes resulting from *KCNJ11* or *ABCC8* mutations, excellent glycaemic control can be achieved with high‐dose sulfonylureas 3, 4, 5.

It remains unclear, however, how sulfonylurea‐treated monogenic diabetes should be managed in pregnancy. The sulfonylurea most widely use in pregnancy is glibenclamide. Until recently it was believed that transfer across the placenta was minimal; however, evidence from its use in gestational diabetes mellitus (GDM) now clearly shows that glibenclamide crosses the placenta 6, 7 and stimulates fetal insulin secretion, resulting in increased fetal growth and increased rates of neonatal hypoglycaemia 8, 9, 10.

In monogenic diabetes the situation is more complex, as there are specific considerations in addition to concern about maternal glycaemia; these include the sulfonylurea doses required, the fetal mutation status and the desirability of exposure to sulfonylureas that differs across the specific genetic subtypes.

In the present review we discuss first the evidence for use of glibenclamide in pregnancies in mothers who do not have monogenic diabetes. We then consider the three common subtypes of sulfonylurea‐treated monogenic diabetes, HNF1A and HNF4A MODY and KCNJ11/ABCC8 permanent neonatal diabetes. We outline sulfonylurea treatment outside pregnancy in these conditions and review the evidence for treatment and outcome in pregnancy. We also suggest a practical approach given the paucity of evidence available.

## Literature search

A systematic literature review was undertaken using PubMed, Embase and OVID. Keywords included: monogenic diabetes, MODY, *HNF1A, HNF4A*, ATP‐sensitive potassium channel (K_ATP_) neonatal diabetes mellitus (*KCNJ11* and *ABCC8*), pregnancy, glibenclamide or glyburide, and gestational diabetes.

## Sulfonylurea treatment in pregnancy in Type 2 diabetes and gestational diabetes: a changing landscape

Data on the use of glibenclamide in pregnancy consist of more than 9500 exposures, mainly in late pregnancy in women with GDM 11. Glibenclamide has until recently been considered a safe drug to use in pregnancy. *In vitro* studies suggested that transfer across the placenta was minimal 12, 13, 14 and glibenclamide was undetectable in cord blood 15. Early clinical studies suggested there was no significant increase in macrosomia or neonatal hypoglycaemia with the use of glibenclamide compared to insulin use 15, 16, 17, 18, 19, 20, but these studies were limited in their power to demonstrate differences 21. In a meta‐analysis (10 studies on 471 women exposed to sulfonylureas and biguanides in the first trimester), no significant difference was found in the rate of major malformations or neonatal death in the offspring of women with exposure to oral antidiabetic agents in the first trimester compared with non‐exposed women, but the meta‐analysis was limited by study heterogeneity 22. In one study of 379 pregnancies there was a significant increase in perinatal mortality (125/1000 births vs 33/1000 births) and stillbirth with oral glucose‐lowering agents vs those treated with insulin (91/1000 births v 33/1000 births; *P*<0.05), but the authors of that study concluded that early exposure to these agents was unlikely to be deleterious 23.

Subsequent studies, using more sensitive assays, have confirmed that glibenclamide does cross the placenta, with umbilical cord plasma concentrations averaging 70% of maternal values 24. More recent publications have demonstrated an increased risk of obstetric and neonatal complications with sulfonylurea use. Even though good glycaemic control may be maintained, pregnant women with GDM treated with glibenclamide had larger babies than insulin‐treated mothers, further increasing the risk of obstetric and neonatal complications from macrosomia 25, 26. In a meta‐analysis of randomized controlled trials, glibenclamide use resulted in greater birth weight (mean difference 109 g; 95% CI 36–181), and higher risks of macrosomia [risk ratio (RR) 2.62, 95% CI 1.35–5.08) and fetal hypoglycaemia (RR 2.04, 95% CI 1.30–3.20) 10. In a large cohort study from the USA in 110 000 women with GDM, newborns of women treated with glibenclamide (glyburide) were at higher risk of neonatal intensive care unit admission (RR 1.41; 95% CI 1.23–1.62), respiratory distress (RR 1.63; 95% CI 1.23–2.15), hypoglycaemia (RR 1.40; 95% CI 1.00–1.95), birth injury (RR 1.35; 95% CI 1.00–1.82), and large for gestational age (RR 1.43, 95% CI 1.16–1.76) compared with those treated with insulin 26. An additional study in 2073 women also found higher odds of neonatal intensive care admission (adjusted odds ratio 1.46, 95%CI 1.07–2.00) and birth weight >4000 g (adjusted odds ratio 1.29, 95% CI 1.03–1.64) amongst infants born to mothers receiving glibenclamide during pregnancy 27. The evidence now indicates that glibenclamide treatment in the third trimester exacerbates fetal hyperinsulinism *in utero* and has an additive effect on birth weight 10, 26, 28. These studies have raised concern regarding the use of glibenclamide in pregnancy 25.

There is limited experience of sulfonylurea use in the first trimester; the data that exist suggest sulfonylureas are not teratogenic when used at conception or in the first trimester 22, 23, 29, although maternal HbA_1c_ was independently associated with congenital anomalies 30. Many studies group women who are on different oral agents together, however, so data for specific sulfonylureas, and in particular glibenclamide, are not available. As there are extremely limited data on other sulfonylureas, and these are not recommended in pregnancy, we do not consider these in the present review.

Current recommendations for the use of glibenclamide in pregnancy now reflect the concerns that have arisen from the recent studies. Glibenclamide is not recommended in GDM if insulin or metformin is available 10, 31. In women with Type 2 diabetes it is recommended that oral antidiabetic agents (other than metformin) are discontinued before pregnancy, and insulin substituted 31. The UK Teratology Information Service (UKTIS) advises, in the absence of evidence for teratogenicity, that glibenclamide may be considered in pregnancy where clinically indicated 11; this could include K_ATP_ neonatal diabetes and *HNF1A*/*HNF4A* MODY, where excellent glycaemic control can be achieved with sulfonylurea treatment. Glibenclamide can be resumed post‐delivery and during breastfeeding for those with pre‐existing diabetes 32.

Current opinion suggests that glibenclamide is not teratogenic, and that it is probably safe in the first trimester. Its use in later pregnancy is a cause for concern, with an increase in adverse fetal outcomes reported, in particular excess fetal growth and neonatal hypoglycaemia.

## What are the specific issues for sulfonylurea‐treated monogenic diabetes in pregnancy ?

Individuals with HNF1A MODY, HNF4A MODY and KCNJ11/ABCC8 permanent neonatal diabetes can achieve excellent control on sulfonylureas outside pregnancy, and glycaemic control may be better on sulfonylureas than insulin 1, 2, 3, 4, 5; however, given that glibenclamide treatment increases the risk of macrosomia and neonatal hypoglycaemia, its use in pregnancy in women with monogenic diabetes needs to be reconsidered.

These conditions are dominantly inherited, so there is a 50% chance of each fetus inheriting the mutation from their affected parent. Sulfonylureas crossing the placenta may be beneficial or detrimental depending on fetal genotype, but in the majority of cases, the fetal genetic status will be unknown, making management more complicated.

**Figure 1:**
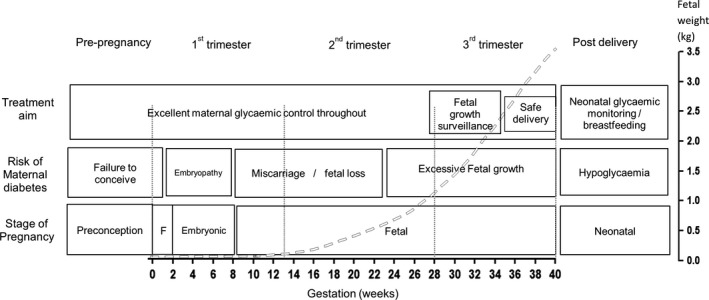
Risks associated with different stages of pregnancy in a mother with diabetes. Dashed line indicates fetal weight gain across advancing gestation and is population‐derived, representing the 50th percentile of fetal growth as the trajectory for normal fetal growth. F, fertilisation.

Treatment decisions need to consider many factors, including monogenic subtype, pre‐pregnancy glycaemic control and treatment, gestation, patient preference, fetal growth and fetal inheritance status (if known). Optimum glycaemic control is essential during organogenesis, and fetal growth is an important consideration with advancing gestation (Fig. 1). Decisions regarding pregnancy management are, of course, individual and should be made with multidisciplinary input involving the patient, diabetologists and obstetricians.

As there are different issues to consider within the three subtypes we will discuss each separately.

## Monogenic diabetes pregnancy

### HNF1A maturity‐onset diabetes of the young

#### Optimum therapy outside pregnancy

HNF1A MODY is optimally managed using low doses of sulfonylureas 1, 2, 33. The mechanism for the increased glycaemic response to sulfonylureas observed in HNF1A MODY, compared with Type 2 diabetes, is thought to be attributable to increased pancreatic insulin secretory response to sulfonylureas and increased sensitivity to the insulin released 1.

#### Reports on pregnancy

##### Impact of fetal genotype

The largest study of neonatal outcomes associated with monogenic diabetes found no difference in birth weight or rate of macrosomia in 134 infants inheriting HNF1A MODY mutations compared with their unaffected siblings (median birth weight 3490 g) 34. Mutations in *HNF1A* were not associated with a greater birth weight, with a median difference of 10 g and a mean difference in the analysis of 24 discordant sibling pairs of 3 g. Neither the birth weight nor the incidence of hypoglycaemia in heterozygous *HNF1A* mutation carriers differed from their unaffected sibling, suggesting that fetal insulin secretion was not increased in *HNF1A* mutation carriers 34. If the mother carried the mutation, the birth weight was increased as a result of maternal hyperglycaemia, but the increase was similar if the fetus was affected or unaffected. Similar findings were identified in other cohorts 35. A single reported case of neonatal hypoglycaemia in a *HNF1A* mutation carrier resolved within 48 h 34. Consequently, inheritance of an *HNF1A* mutation is not considered to be associated with adverse birth outcomes or obstetric morbidity.

##### Impact of maternal therapy in pregnancy

There are no reports on the outcomes of sulfonylurea treatment in HNF1A MODY pregnancies, so the neonatal effects of sulfonylureas are not known.

#### Recommendations

**Figure 2:**
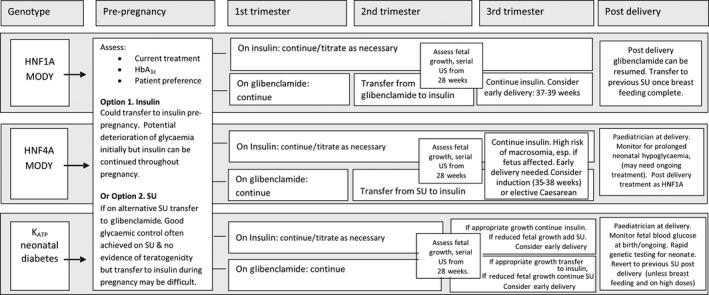
Options for pregnancy management in HNF1A/HNF4A maturity‐onset diabetes of the young (MODY) and ATP‐sensitive potassium (K_ATP_) channel neonatal diabetes. SU, sulfonylurea; US, ultrasonography.

There are two main treatment options for HNF1A MODY in pregnancy (Fig. 2): stop sulfonylureas pre‐pregnancy and transfer to insulin or treat with glibenclamide pre‐/early pregnancy and transfer to insulin in the second trimester. The second option should only be considered if pre‐pregnancy HbA_1c_ reaches local targets for glycaemic control. The advantage of excellent glycaemic control at conception and in the first trimester needs to be balanced against the lack of sufficient safety data on glibenclamide in the first trimester and the need to transfer from glibenclamide before the third trimester. Using insulin prior to conception has the advantage that this treatment can be continued throughout pregnancy and has a proven safety record at all stages of pregnancy; however, optimum glycaemic control at the time of conception and in the first trimester may be harder to achieve with insulin therapy.

If a woman taking sulfonylureas presents already pregnant, the benefit of transferring to insulin in early pregnancy needs to be weighed up against the potential deterioration in glycaemic control during organogenesis. In women with good glycaemic control [HbA_1c_ < 48 mmol/mol (6.5%)] on a sulfonylurea, the deterioration in control on discontinuing a sulfonylurea can be marked, and there is a case for continuing with a sulfonylurea until the end of the first trimester. Those on an alternate sulfonylurea should be transferred to an equivalent dose of glibenclamide.

If used before pregnancy and in the first trimester, glibenclamide should be discontinued before the third trimester, to avoid its trans‐placental transfer. If this option is chosen, we suggest introducing a basal insulin in the second trimester and then replacing glibenclamide with bolus insulin as a second phase, aiming to achieve excellent glycaemic control well before week 26 of gestation. If glibenclamide is continued into the final trimester, doses should be as low as possible (≤5 mg/day) as the impact on the fetus is likely to be dose‐related.

We recommend that women with HNF1A MODY have the same fetal checks/scans as recommended for other pre‐existing diabetes in pregnancy 31. Delivery should be considered between 37 and 38^+6^ weeks, in line with management of other pregnancies involving pre‐existing diabetes 31. Glibenclamide can be resumed post‐delivery and during breastfeeding, with transfer, if desired, to an alternative sulfonylurea after weaning.

### HNF4A maturity‐onset diabetes of the young

#### Optimum therapy outside pregnancy

Low‐dose sulfonylureas are the optimum therapy in HNF4A MODY outside pregnancy 2.

#### Reports on pregnancy

##### Impact of fetal genotype

Inheritance of an *HNF4A* mutation results in a dramatic increase in birth weight. In a study of 108 individuals from families with HNF4A MODY, babies inheriting an *HNF4A* mutation had a median birth weight 790 g greater than their unaffected siblings (97^th^ v 58^th^ centile) 34. There was an additional effect of maternal glycaemia; hence affected *HNF4A* infants born to mothers with HNF4A MODY had a median corrected birth weight of 4840 g compared with 4170 g when the father was affected 34. The prevalence of macrosomia was 64% if the *HNF4A* mutation was inherited from the mother and 46% if it was inherited from the father. Extreme macrosomia (birth weight >5000 g) was seen in 15% of cases with an affected mother and 7% of cases with an affected father 34. The increase in birth weight when the mother is affected is likely to be attributable to the effects of maternal hyperglycaemia.

The increased fetal growth associated with inheriting a fetal *HNF4A* mutation results in marked morbidity including neonatal hypoglycaemia, shoulder dystocia, brachial plexus birth injury, assisted delivery and emergency caesarean section 34, 36, 37, 38, 39. Infants born to a parent with HNF4A MODY require glucose monitoring after delivery as at least 10% of affected neonates have hypoglycaemia (blood glucose <2.5 mmol/l) 34, 40. Severe hyperinsulinaemic hypoglycaemia (blood glucose 0.8–2.5mmol/l for >24 h) may require prolonged treatment (intravenous glucose infusion, glucagon and diazoxide/chlorthiazide) as the hypoglycaemia can persist for months or years 37, 40, 41. The increased birth weight and neonatal hypoglycaemia are a result of hyperinsulinism before and after birth 34. The only exception is found in individuals with the p.R114W mutation, which is atypical of HNF4A MODY in that it has no effect on birth weight 42.

##### Impact of maternal therapy in pregnancy

Reports of maternal treatment during pregnancy in HNF4A MODY are scarce. In one case, insulin treatment did not prevent macrosomia or hypoglycaemia 38. The published data are inadequate to determine if there is an effect of sulfonylurea use.

#### Recommendations

As there is a very high risk of macrosomia in *HNF4A* pregnancies if the fetus is affected, achieving excellent glycaemic control is essential. The two main treatment options (Fig. 2) are the same as those for HNF1A pregnancies, as discussed above. Additional information specific to *HNF4A* pregnancies is detailed below.

In women with *HNF4A* MODY, serial growth assessment should be undertaken from 28 weeks, at 2‐weekly intervals at least, depending on growth trajectory, in addition to routine anomaly screening at earlier gestation as advised by the National Institute of Health and Care Excellence (NICE), to detect developing macrosomia in affected fetuses. Early delivery is needed even with excellent glucose control if the fetus is genetically affected. Induction of labour or elective caesarean section should be considered from 35 to 38 weeks, based on estimated fetal size on ultrasonography. A paediatrician should be available at delivery. Post‐delivery the infant should be monitored for neonatal hypoglycaemia for at least 48 h, as this may be prolonged and need continued treatment. The mother can resume glibenclamide post‐delivery and during breastfeeding, with transfer to an alternative sulfonylurea, if desired, once breastfeeding is completed.

#### Management of pregnancies when the father has HNF4A MODY

A fetus inheriting the *HNF4A* mutation from the father has a similar risk of macrosomia and its complications (shoulder dystocia, obstructed birth, etc) to that associated with maternal inheritance (median corrected birth weight 4200 g) 34. In addition, there is a high risk of fetal hypoglycaemia that may be severe and profound (see above). We therefore recommend regular ultrasonography monitoring from 28 weeks’ gestation if the father has HNF4A MODY. If there is evidence of fetal macrosomia on ultrasonography, early delivery (37–38^+6^ weeks) should be considered. A paediatrician should review the child soon after birth and assess for hypoglycaemia (see recommendations above).

### K_ATP_ neonatal diabetes

#### Optimum therapy outside pregnancy

The vast majority of individuals with KCNJ11 or ABCC8 permanent neonatal diabetes are successfully managed with glibenclamide, with improvements in HbA1c and no increase in hypoglycaemia 3, 4, 43. The key difference in sulfonylurea treatment in neonatal diabetes is the high dose of glibenclamide required (0.45 mg/kg/day) 3 compared with the low doses in HNF1A/HNF4A MODY (<0.01 mg/kg/day) 1, 2 or typical doses in Type 2 diabetes (0.06–0.2 mg/kg/day) 32.

#### Reports on pregnancy

##### Impact of fetal genotype

In K_ATP_ neonatal diabetes pregnancies neonatal birth weight is dependent on fetal genotype. If the fetus has inherited the genetic mutation from either the mother or father, reduced fetal insulin secretion results in low birth weight (median 2580 g at median 39 weeks’ gestation) 44, 45, 46.

##### Impact of maternal therapy in pregnancy

Glibenclamide treatment may be beneficial or detrimental, depending on whether or not the fetus is affected. Three women with *KCNJ11* mutations who were treated with a sulfonylurea during four pregnancies have been described. The glibenclamide doses ranged from 2.8 to 90 mg/day 47, 48, 49. If the fetus is unaffected, then maternal use of high‐dose glibenclamide leads to high fetal doses, excess fetal insulin secretion, excess insulin‐mediated growth and neonatal hypoglycaemia 47, 48, 49. In contrast when the fetus is affected, trans‐placental transfer of sulfonylurea restores fetal K_ATP_ function and improves fetal growth, resulting in normal birth weight (median 3010 g at 38 weeks) 47. Interestingly, one of the children described has not presented with neonatal diabetes in the first 18 months of life 47. Whether this is a legacy effect of the high‐dose glibenclamide or a mutation effect (the E229K mutation causes transient neonatal diabetes) 47 is not clear.

Offspring (*n*=6) who inherited a *KCNJ11* mutation from their affected insulin‐treated mothers had a normal birth weight. In contrast, if the baby was born to a non‐diabetic mother, birth weight was reduced (−1.81 v −0.12 median Standard Deviation Score for birth weight) 45. This suggests that maternal hyperglycaemia can lead to increased fetal growth, even when there is a fetal mutation greatly reducing fetal insulin secretion.

##### Breastfeeding

In one mother with *KCNJ11* neonatal diabetes taking very high doses of glibenclamide (90 mg a day), persistent postnatal exposure led to hypoglycaemia in her unaffected neonate through transfer of the drug in breast milk 48; however, the doses of glibenclamide typically used in Type 2 diabetes are considered safe for breastfeeding 32.

#### Recommendations

The majority of women with K_ATP_ neonatal diabetes will already be treated with glibenclamide pre‐pregnancy and excellent glycaemic control can usually be achieved [typically HbA_1c_ <48 mmol/mol (6.5%)]. If it is decided to continue sulfonylureas pre‐conception and in the first trimester to aid glycaemic control then it is appropriate to reduce the glibenclamide dose to the lowest that maintains HbA_1c_ concentration at ≤48 mmol/mol (6.5%).

If pregnancy is planned, transfer to insulin may be considered pre‐conception but this will usually result in deterioration of glycaemic control with potential consequences with regard to fetal outcome.

The fetal genotype has a crucial role in determining whether glibenclamide therapy is recommended in K_ATP_ neonatal diabetes. To determine if the fetus is affected, fetal genotyping should be offered if amniocentesis or chorionic villus sampling are being performed for another reason. Some authors have suggested that fetal genetic testing should be performed in all affected mothers, despite the risk (~1%) of miscarriage associated with invasive testing, as it would directly alter management 48, 49. If the fetal genotype is not tested directly, it may be possible to infer whether a fetus is likely to be affected by detecting reduced fetal growth by serial ultrasonography from 28 weeks’ gestation.

If the fetus is affected, maternal glibenclamide treatment is appropriate when the mother has K_ATP_ neonatal diabetes, as it will cross the placenta and provide *in utero* treatment of an affected fetus. This improves both fetal growth and, potentially, brain development. If the fetus is thought to be affected, glibenclamide should be continued at the lowest dose required for optimum glycaemic control. If the mother had been transferred to insulin pre‐conception then glibenclamide should be reintroduced at the pre‐pregnancy dose. If the mutation has been inherited from an affected father, treatment with sulfonylurea during pregnancy is not possible.

If the fetus is unaffected, or there is no ultrasound evidence that it is affected, glibenclamide in the last trimester will exacerbate excessive fetal growth and neonatal hypoglycaemia, therefore, insulin is advised; however, transfer to insulin will risk deterioration in glycaemic control at this time. Transfer from glibenclamide to insulin should be rapid (<3 days), a basal‐bolus regimen should be introduced as the glibenclamide is stopped and rapidly titrated to achieve good glycaemic control.

Timing of delivery should take into account the risk of continuing pregnancy in a suboptimum intrauterine growth environment vs that of prematurity from early intervention. Usually this will be between 37 and 38^+6^ weeks.

A fetus inheriting a K_ATP_ mutation from its mother or father will usually present with diabetes before the age of 6 months and will have elevated glucose levels before presenting with ketoacidosis. Prompt diagnosis and treatment is essential. A paediatrician should be available at delivery and the neonate's blood glucose should be checked at birth and at least 8‐hourly for a minimum of 48 h. If the fetal genotype was not determined in pregnancy, rapid genetic testing of the fetus should be offered, as 50% of cases will be affected.

Cord blood samples can be sent to the molecular genetics laboratory at the Royal Devon and Exeter NHS Foundation Trust (http://www.diabetesgenes.org) and will be tested as a priority, without charge. Genetic test results in these cases should be available within 1 week of the arrival of the sample. The initial treatment of neonatal diabetes should usually be insulin, but sulfonylurea can be started as soon as the diagnosis of KATP neonatal diabetes is confirmed.

After delivery, women with K_ATP_ neonatal diabetes can restart glibenclamide at pre‐pregnancy doses. The exception to this is when doses >0.2 mg/kg/day are used and breastfeeding is planned. The transfer of the drug in breast milk may be significant at higher doses 48.

## Antenatal testing for fetal genetic diagnosis

Awareness of fetal genotype allows individualized obstetric care, but fetal genotype is usually unknown, as at present it requires invasive testing. Due to the risks involved in chorionic villus sampling or amniocentesis, genetic testing is only usually considered antenatally if these tests are performed for another reason. Fetal growth on ultrasonography may provide a surrogate of fetal inheritance in HNF4A MODY and K_ATP_ neonatal diabetes in the absence of a confirmatory antenatal genetic test, and may be useful in guiding the most appropriate management. Non‐invasive genetic testing of cell‐free fetal DNA in maternal blood is likely to revolutionize the management of monogenic diabetes pregnancies in the future 50.

## Conclusions

Glibenclamide is now known to cross the placenta and its use in pregnancy can result in increased risk of large‐for‐gestational‐age infants, macrosomia and neonatal hypoglycaemia. The management of monogenic diabetes pregnancies that are treated with sulfonylureas outside pregnancy aims to achieve optimum glycaemic control during the first trimester, whilst limiting the adverse effects of glibenclamide on fetal birth weight.

We recommend avoiding glibenclamide use during the third trimester in women with HNF1A/HNF4A MODY. In K_ATP_ neonatal diabetes glibenclamide can be continued throughout pregnancy if the fetus has reduced or low/normal fetal growth or is known to have inherited a K_ATP_ channel mutation.

In the future, non‐invasive pre‐natal genetic testing will enable personalized management of HNF4A MODY and K_ATP_ neonatal diabetes pregnancies. As data are currently lacking, we advocate an international system of reporting all pregnancy management/outcomes in monogenic diabetes pregnancies to enable an evidence‐based approach. The monogenic diabetes team at the Royal Devon and Exeter NHS Foundation Trust would offer to act as a repository collating this information.

## Funding sources

Primary funding (for salary support) was received from the Wellcome Trust and National Institute for Health Research (NIHR)

## Competing interests

None declared.

## References

[1] Pearson ER , Starkey BJ , Powell RJ , Gribble FM , Clark PM , Hattersley AT . Genetic cause of hyperglycaemia and response to treatment in diabetes. Lancet 2003; 362: 1275–1281. 14575972 10.1016/S0140-6736(03)14571-0

[2] Pearson ER , Pruhova S , Tack CJ , Johansen A , Castleden HA , Lumb PJ *et al* Molecular genetics and phenotypic characteristics of MODY caused by hepatocyte nuclear factor 4alpha mutations in a large European collection. Diabetologia 2005; 48: 878–885. 15830177 10.1007/s00125-005-1738-y

[3] Pearson ER , Flechtner I , Njolstad PR , Malecki MT , Flanagan SE , Larkin B *et al* Switching from insulin to oral sulfonylureas in patients with diabetes due to Kir6.2 mutations. N Engl J Med 2006; 355: 467–477. 16885550 10.1056/NEJMoa061759

[4] Rafiq M , Flanagan SE , Patch AM , Shields BM , Ellard S , Hattersley AT . Effective treatment with oral sulfonylureas in patients with diabetes due to sulfonylurea receptor 1 (SUR1) mutations. Diabetes Care 2008; 31: 204–209. 18025408 10.2337/dc07-1785 PMC7611807

[5] Klupa T , Skupien J , Mirkiewicz‐Sieradzka B , Gach A , Noczynska A , Zubkiewicz‐Kucharska A *et al* Efficacy and safety of sulfonylurea use in permanent neonatal diabetes due to KCNJ11 mutations: 34‐month median follow‐up. Diabetes Technol Ther 2010; 12: 387–391. 20184447 10.1089/dia.2009.0165

[6] Ryu RJ , Hays KE , Hebert MF . Gestational diabetes mellitus management with oral hypoglycaemic agents. Semin Perinatol 2014; 38: 508–515. 25315294 10.1053/j.semperi.2014.08.012 PMC4252887

[7] Schwartz RA , Rosenn B , Aleksa K , Koren G . Glyburide transport across the human placenta. Obstet Gynecol. 2015; 125: 583–588. 25730219 10.1097/AOG.0000000000000672

[8] Poolsup N , Suksomboon N , Amim M . Efficacy and safety of oral antidiabetic drugs in comparison to insulin in treating gestational diabetes mellitus: a meta‐analysis. PLoS One 2014; 9: e109985. 25302493 10.1371/journal.pone.0109985 PMC4193853

[9] Zeng YC , Li MJ , Chen Y , Jiang L , Wang SM , Mo XL *et al* The use of glyburide in the management of gestational diabetes mellitus: a meta‐analysis. Adv Med Sci 2014; 59: 95–101. 24797983 10.1016/j.advms.2014.03.001

[10] Balsells M , Garcia‐Patterson A , Sola I , Roque M , Gich I , Corcoy R . Glibenclamide, metformin, and insulin for the treatment of gestational diabetes: a systematic review and meta‐analysis. BMJ 2015; 350: h102. 25609400 10.1136/bmj.h102 PMC4301599

[11] United Kingdom Teratology Information Service (UKTIS) . Use of Glibenclamide in pregnancy. Available at http://www.medicinesinpregnancy.org/bumps/monographs/USE-OF-GLIBENCLAMIDE-IN-PREGNANCY/. Last accessed 28 October 2016.

[12] Elliott BD , Langer O , Schenker S , Johnson RF . Insignificant transfer of glyburide occurs across the human placenta. Am J Obstet Gynecol 1991; 165: 807–812. 1951536 10.1016/0002-9378(91)90421-m

[13] Koren G . Glyburide and fetal safety; transplacental pharmacokinetic considerations. Reprod Toxicol 2001; 15: 227–229. 11390165 10.1016/s0890-6238(01)00122-8

[14] Garcia‐Bournissen F , Feig DS , Koren G . Maternal‐fetal transport of hypoglycaemic drugs. Clin Pharmacokinet. 2003; 42: 303–313. 12648023 10.2165/00003088-200342040-00001

[15] Langer O , Conway DL , Berkus MD , Xenakis EM , Gonzales O . A comparison of glyburide and insulin in women with gestational diabetes mellitus. N Engl J Med 2000; 343: 1134–1138. 11036118 10.1056/NEJM200010193431601

[16] Lim JM , Tayob Y , O'Brien PM , Shaw RW . A comparison between the pregnancy outcome of women with gestation diabetes treated with glibenclamide and those treated with insulin. Med J Malaysia 1997; 52: 377–381. 10968114

[17] Moretti ME , Rezvani M , Koren G . Safety of glyburide for gestational diabetes: a meta‐analysis of pregnancy outcomes. Ann Pharmacother 2008; 42: 483–490. 18349305 10.1345/aph.1K577

[18] Jacobson GF , Ramos GA , Ching JY , Kirby RS , Ferrara A , Field DR . Comparison of glyburide and insulin for the management of gestational diabetes in a large managed care organization. Am J Obstet Gynecol 2005; 193: 118–24. 16021069 10.1016/j.ajog.2005.03.018

[19] Rochon M , Rand L , Roth L , Gaddipati S . Glyburide for the management of gestational diabetes: risk factors predictive of failure and associated pregnancy outcomes. Am J Obstet Gynecol 2006; 195: 1090–1094. 17000241 10.1016/j.ajog.2006.07.029

[20] Holt RIG , Clarke P , Parry EC , Coleman MAG . The effectiveness of glibenclamide in women with gestational diabetes. Diabetes Obes Metab 2008; 10: 906–911. 18093212 10.1111/j.1463-1326.2007.00828.x

[21] Rand L , Caughey AB . Comment on ‘Comparison of glyburide and insulin for the management of gestational diabetes in a large managed care organization. Am J Obstet Gynecol 2006; 195: 628–629. 16579949 10.1016/j.ajog.2005.11.016

[22] Gutzin SJ , Kozer E , Magee LA , Feig DS , Koren G . The safety of oral hypoglycemic agents in the first trimester of pregnancy: a meta‐analysis. Can J Clin Pharmacol 2003; 10: 179–183. 14712322

[23] Ekpebegh CO , Coetzee EJ , van der Merwe L , Levitt NS . A 10‐year retrospective analysis of pregnancy outcome in pregestational Type 2 diabetes: comparison of insulin and oral glucose‐lowering agents. Diabet Med 2007; 24: 253–258. 17305787 10.1111/j.1464-5491.2007.02053.x

[24] Hebert MF , Ma X , Naraharisetti SB , Krudys KM , Umans JG , Hankins GD *et al* Are we optimizing gestational diabetes treatment with glyburide? The pharmacologic basis for better clinical practice. Clin Pharmacol Ther 2009; 85: 607–614. 19295505 10.1038/clpt.2009.5 PMC2684566

[25] Holt RIG . Glyburide for gestational diabetes: time for a pause for thought. JAMA Pediatr 2015; 169: 427–428. 25821923 10.1001/jamapediatrics.2015.144

[26] Camelo Castillo W , Boggess K , Sturmer T , Brookhart MA , Benjamin DK , Jonsson Funk M . Association of adverse pregnancy outcomes with glyburide vsiInsulin in women with gestational diabetes. JAMA Pediatr 2015; 169: 452–458. 25822253 10.1001/jamapediatrics.2015.74

[27] Cheng YW , Chung JH , Block‐Kurisch I , Inturrisi M , Caughey AB . Treatment of gestational diabetes mellitus: glyburide comnpared to subcutaneous insulin therapy and associated perinatal outcomes. J Matern Fetal Neonatol 2012; 25: 379–384. 10.3109/14767058.2011.580402 PMC3443974 21631239

[28] Amin M , Suksomboon N , Poolsup N , Malik O . Comparison of glyburide with metformin in treating gestational diabetes mellitus: a systematic review and meta‐analysis. Clin Drug Invest 2015; 35: 343–351. 10.1007/s40261-015-0289-3 25985837

[29] Towner D , Kjos S , Leung B , Montoro MM , Xiang A , Mestman JH *et al* Congenital malformations in pregnancies complicated by NIDDM. Diabetes Care 1995; 18: 1446–1451. 8722068 10.2337/diacare.18.11.1446

[30] Kaira B , Gupta Y , Singla R , Kaira S . Use of Oral Anti‐Diabetic Agents in Pregnancy: A Pragmatic Approach. N Am J Med Sci 2015; 7: 6–12. 25709972 10.4103/1947-2714.150081 PMC4325398

[31] National Institute of Clinical Excellence (NICE) . Diabetes in pregnancy: management of diabetes and its complications from preconception to the postnatal period. 2015; Feb. National Institute for Health and Care Excellence: Clinical Guidelines. London, UK. Available at https://www.nice.org.uk/guidance/ng3. Last accessed 3 November 2016.

[32] British National Formulary (BNF) 2016; Drugs used in diabetes. Available at https://www.evidence.nhs.uk/formulary/bnf/current/6-endocrine-system/61-drugs-used-in-diabetes/612-antidiabetic-drugs#PHP4128. Last accessed 28 October 2016.

[33] Bacon S , Kyithar MP , Rizvi SR , Donnelly E , McCarthy A , Burke M *et al* Successful maintenance on sulfonylurea therapy and low diabetes complication rates in a HNF1A‐MODY cohort. Diabet Med 2016; 33: 976–984. 26479152 10.1111/dme.12992

[34] Pearson ER , Boj SF , Steele AM , Barrett T , Stals K , Shield JP *et al* Macrosomia and hyperinsulinaemic hypoglycaemia in patients with heterozygous mutations in the HNF4A gene. PLoS Med 2007; 4: e118. 17407387 10.1371/journal.pmed.0040118 PMC1845156

[35] Bacon S , Schmid J , McCarthy A , Edwards J , Fleming A , Kinsley B *et al* The clinical management of hyperglycaemia in pregnancy complicated by maturity onset diabetes of the young. Am J Obstet Gynecol 2015; 213: e1–7. 10.1016/j.ajog.2015.04.037 25935773

[36] Fajans SS , Bell GI . Macrosomia and neonatal hypoglycaemia in RW pedigree subjects with a mutation (Q268X) in the gene encoding hepatocyte nuclear factor 4α (HNF4α). Diabetologia 2007; 50: 2600–2601. 17891372 10.1007/s00125-007-0833-7

[37] Kapoor RR , Locke J , Colclough K , Wales J , Conn JJ , Hattersley AT *et al* Persistent hyperinsulinemic hypoglycemia and maturity‐onset diabetes of the young due to heterozygous HNF4A mutations. Diabetes 2008; 57: 1659–1663. 18268044 10.2337/db07-1657

[38] Conn JJ , Simm PJ , Oats JJN , Nankervis AJ , Jacobs SE , Ellard S *et al* Neonatal hyperinsulinaemic hypoglycaemia and monogenic diabetes due to a heterozygous mutation of the HNF4A gene. Aust N Z J Obstet Gynaecol 2009; 49: 328–339. 19566570 10.1111/j.1479-828X.2009.01009.x

[39] Colombo C , Geraci C , Suprani T , Pocecco M , Barbetti F . Macrosomia, transient neonatal hypoglycaemia and monogenic diabetes in a family with heterozygous mutation R154X of the HNF4A gene. J Endocrinol Invest 2011; 34: 252–253. 21537110 10.1007/BF03347074

[40] Flanagan SE , Kapoor RR , Mali G , Cody D , Murphy N , Schwahn B *et al* Diazoxide‐responsive hyperinsulinemic hypoglycemia caused by HNF4A gene mutations. Eur J Endocrinol 2010; 162: 987–992. 20164212 10.1530/EJE-09-0861 PMC2857991

[41] McGlacken‐Byrne SM , Hawkes CP , Flanagan SE , Ellard S , McDonnell CM , Murphy NP . The evolving course of HNF4A hyperinsulinaemic hypoglycaemia–a case series. Diabet Med 2014; 31: e1–5. 23796040 10.1111/dme.12259

[42] Laver TW , Colclough K , Shepherd M , Patel K , Houghton JAL , Dusatkova P *et al* The common p. R114W HNF4A mutation causes a distinct clinical subtype of monogenic diabetes. Diabetes 2016; 65: 3212–3217. 27486234 10.2337/db16-0628 PMC5035684

[43] Sagen JV , Raeder H , Hathout E , Shehadeh N , Gudmundsson K , Baevre H *et al* Permanent neonatal diabetes due to mutations in KCNJ11 encoding Kir6.2: patient characteristics and initial response to sulfonylurea therapy. Diabetes 2004; 53: 2713–2718. 15448106 10.2337/diabetes.53.10.2713

[44] Gloyn AL , Pearson ER , Antcliff JF , Proks P , Bruining GJ , Slingerland AS *et al* Activating mutations in the gene encoding the ATP‐sensitive potassium‐channel subunit Kir6.2 and permanent neonatal diabetes. N Engl J Med 2004; 350:1838–1849. Erratum in: N Engl J Med 2004; 351:1470. 15115830 10.1056/NEJMoa032922

[45] Slingerland AS , Hattersley AT . Activating mutations in the gene encoding Kir6.2 alter fetal and postnatal growth and also cause neonatal diabetes. J Clin Endocrinol Metab 2006; 91: 2782–2788. 16636122 10.1210/jc.2006-0201

[46] Flanagan SE , Edghill EL , Gloyn AL , Ellard S , Hattersley AT . Mutations in KCNJ11, which encodes Kir6.2, are a common cause of diabetes diagnosed in the first 6 months of life, with the phenotype determined by genotype. Diabetologia 2006; 49: 1190–1197. 16609879 10.1007/s00125-006-0246-z

[47] Gaal Z , Klupa T , Kantor I , Mlynarski W , Albert L , Tolloczko J *et al* Sulfonylurea use during entire pregnancy in diabetes because of KCNJ11 mutation: a report of two cases. Diabetes Care 2012; 35: e40. 22619292 10.2337/dc12-0163 PMC3357257

[48] Myngheer N , Allegaert K , Hattersley A , McDonald T , Kramer H , Ashcroft FM *et al* Fetal macrosomia and neonatal hyperinsulinemic hypoglycemia associated with transplacental transfer of sulfonylurea in a mother with KCNJ11‐related neonatal diabetes. Diabetes Care 2014; 37: 3333–3335. 25231897 10.2337/dc14-1247 PMC5894804

[49] Klupa T , Kozek E , Nowak N , Cyganek K , Gach A , Milewicz T *et al* The first case report of sulfonylurea use in a woman with permanent neonatal diabetes mellitus due to KCNJ11 mutation during a high‐risk pregnancy. J Clin Endocrinol Metab 2010; 95: 3599–3604. 20466780 10.1210/jc.2010-0096

[50] De Franco E , Caswell R , Houghton JA , Iotova V , Hattersley AT , Ellard S . Analysis of cell‐free fetal DNA for non‐invasive prenatal diagnosis in a family with neonatal diabetes. Diabet Med 2017; 34: 582–585. 27477181 10.1111/dme.13180 PMC5096683

